# Ferroptosis: a novel perspective on tumor immunotherapy

**DOI:** 10.3389/fimmu.2025.1524711

**Published:** 2025-04-07

**Authors:** Yuan Xu, Mingpai Ge, Yuqing Xu, Kai Yin

**Affiliations:** Changhai Hospital, Second Military Medical University, Shanghai, China

**Keywords:** ferroptosis, immunotherapy, metabolism, tumor immunotherapy, immune cell

## Abstract

Ferroptosis is a novel form of programmed cell death characterized by iron-dependent accumulation of reactive oxygen species (ROS) and lipid peroxidation. The execution of ferroptosis is intricately linked to both iron and lipid metabolism. Intriguingly, iron and lipid metabolism are also pivotal for maintaining the physiological function of immune cells. Research has revealed that ferroptosis can potentiate the immunogenicity of tumor cells and engage in intricate interactions with immune cells. Certain ferroptosis inducers have the capacity to augment the efficacy of immunotherapy by modulating the tumor immune microenvironment. Ferroptosis holds immense potential in cancer immunotherapy and is anticipated to emerge as a novel therapeutic target in the future landscape of cancer treatment. In this review, we primarily delineate the ferroptosis signaling pathways and metabolic processes pertinent to immune cells, and further summarize the roles of ferroptosis in tumor-infiltrating immune cells. Ultimately, we anticipate further elucidation of the mechanisms of ferroptosis in immunotherapy and envision that strategies targeting ferroptosis and immunotherapy will be expeditiously applied in clinical oncology practice.

## 1 Introduction

Programmed cell death, owing to its precisely gene-regulated nature, has become a primary focus in contemporary cancer therapy research (1, 2). Ferroptosis, as a novel mode of programmed cell death discovered in 2012, has progressively emerged as a significant direction in cancer treatment research. The mechanism of ferroptosis is characterized by its dependence on intracellular iron and the accumulation of lipid reactive oxygen species (ROS) (3). Distinct from other forms of programmed cell death, ferroptosis render it considerable potential for development in cancer therapeutics (3, 4). The accumulation of intracellular iron, induced lipid peroxidation, and the failure of the antioxidant defense system not only alter the expression of genes regulating iron metabolism and lipid peroxidation consequently modifying ferroptosis pathway-related genes, but also lead to mitochondrial condensation, increased bilayer membrane density, and subsequent organelle dysfunction (5, 6).Intriguingly, several drugs previously established in clinical practice, such as sorafenib, lorazepam, and artemisinin, have been recently found to exert anti-tumor effects by inducing ferroptosis in cancer cells (7–10).

Cancer immunotherapy, as an emerging paradigm in oncologic therapeutics, exerts its primary mechanism through potentiating the activation of the immune system to target and eradicate malignant cells (11, 12). Due to its favorable anti-tumor efficacy, it has become a mainstay in the clinical management of various solid tumors, including pancreatic carcinoma, mammary carcinoma, ovarian carcinoma, and non-small cell lung carcinoma (13–17). Concurrently, with the progressive deepening of research into cancer immunotherapy, the significance of the interplay between ferroptosis and immunotherapy in cancer treatment is becoming increasingly recognized. Activation of CD8+ T lymphocytes has demonstrated synergistic effects with radiotherapy in suppressing SLC7A11 expression, thereby diminishing cystine uptake—a process that further promotes lipid peroxidation and ferroptosis. These combined effects ultimately augment radiosensitivity (18). Tumor-associated macrophages (TAMs), upon polarization into the M1 macrophage phenotype, can initiate Fenton reaction-mediated ferroptosis, exhibiting synergistic activity in conjunction with PD-1 antibodies and TGF-β inhibitors (19). Conversely, CD36-mediated ferroptosis has been shown to impede CD8(+) T cell effector function, thereby attenuating their anti-tumor capacity (20). Given the substantial therapeutic potential inherent in both ferroptosis and immunotherapy for cancer management, this review aims to synthesize the current understanding of their functional roles and intricate interrelationships in oncotherapeutic applications. This review is to delineate potential avenues for future research and therapeutic strategies through the pivotal mechanisms and recent advancements of ferroptosis.

## 2 Signaling pathways in ferroptosis

### 2.1 GPX4 pathway

Glutathione peroxidase 4 (GPX4) indisputably holds a central and pivotal position within the intricate regulatory architecture of ferroptosis. Functioning as a critical intracellular enzymatic entity, GPX4 executes an indispensable role by catalyzing the reductive conversion of lipid hydroperoxides (L-OOH) into benign lipid alcohols (L-OH) (21). Empirical investigations have unequivocally demonstrated that the profound depletion of glutathione (GSH) levels precipitates a direct abrogation of GPX4 enzymatic functionality. Conversely, the meticulous modulation of GPX4 expression levels, encompassing both transcriptional upregulation and downregulation, exerts a demonstrably significant influence on the vulnerability of neoplastic cells to ferroptosis-inducing agents. These compelling findings furnish irrefutable evidence that GPX4 constitutes a cardinal determinant governing neoplastic cell demise, specifically via the ferroptosis pathway (22, 23). Consequently, the GPX4-centric ferroptosis regulatory axis emerges as a highly promising trajectory for the innovative design and development of targeted therapeutics in oncological intervention.

**Figure 1:**
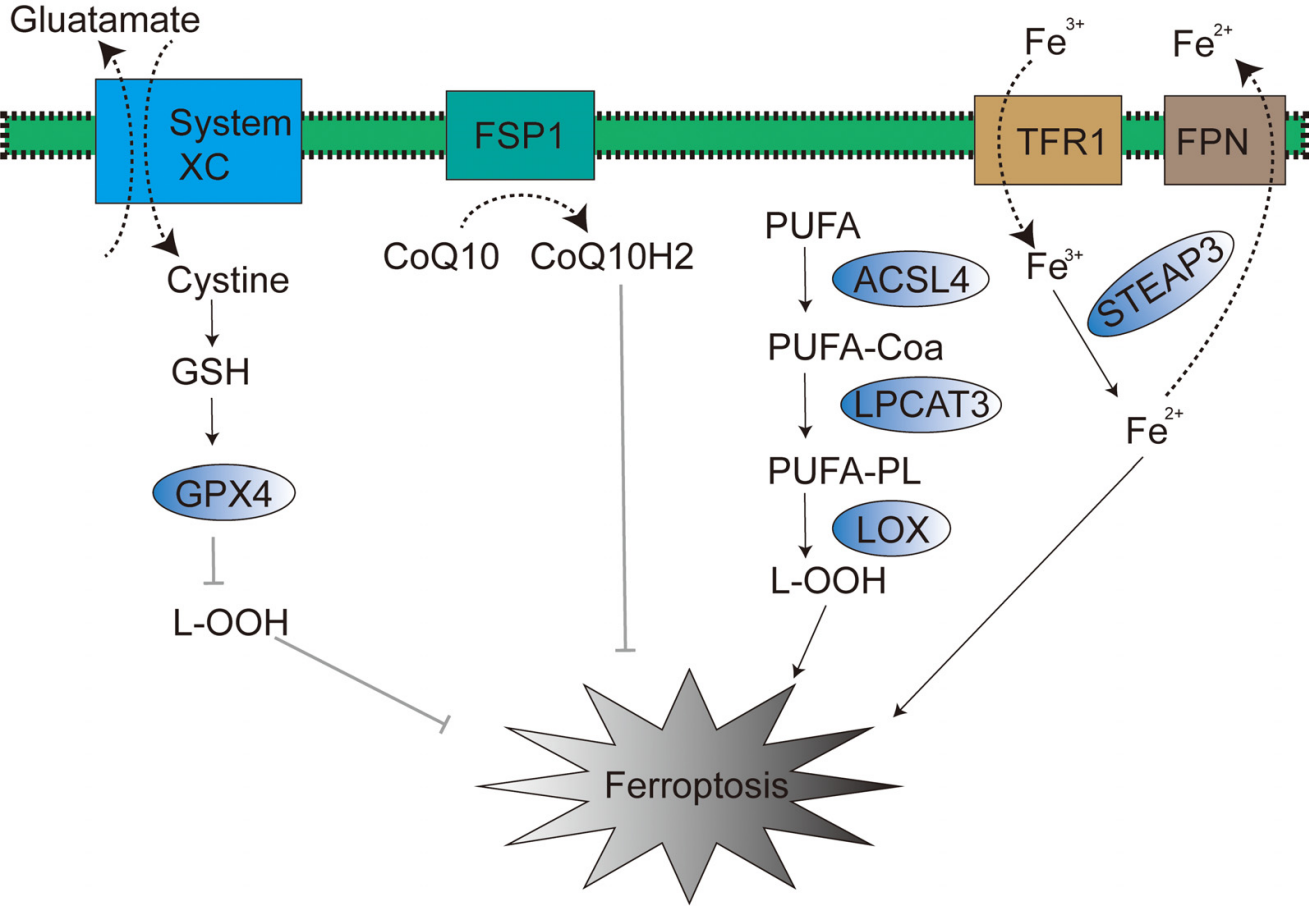
Signaling pathways in ferroptosis.

The uncompromised expression and efficacious physiological function of GPX4 are rigorously contingent upon the synergistic biochemical partnership between selenium and glutathione (GSH) (24). Selenium and GSH are not merely endogenous and indispensable key constituents in the cellular orchestration of ferroptosis, a fundamentally crucial biological process, but also operate as obligatory cofactors essential for GPX4 enzyme to execute its canonical catalytic activity. Intracellular GSH biosynthesis is intricately and obligately reliant upon the bio-availability of the amino acid cystine. The cellular acquisition of intracellular free cystine is predominantly and actively mediated by the cystine/glutamate antiporter system (system Xc-), a transmembrane transport apparatus abundantly expressed on the cellular membrane (**Figure 1**). Subsequent to cytoplasmic entry, cystine undergoes rapid enzymatic conversion to cysteine, thereby serving as an indispensable metabolic precursor in the GSH biosynthetic cascade. Of particular note, cysteine unequivocally stands as the cardinal rate-limiting amino acid for GSH biosynthesis, and its intracellular bioavailability exerts direct governance over GSH synthetic efficiency, thereby intrinsically modulating GPX4 physiological function and the execution of ferroptosis.

The System Xc- transporter manifests as a heterodimeric proteinaceous complex, functionally and structurally constituted by two obligate subunits, SLC7A11 and SLC3A2, which operate in a tightly coordinated and synergistic manner. And tumor suppressor gene TP53 (encoding p53 protein) exerts robust negative regulatory command over the transcriptional expression of the SLC7A11 subunit (25). This precise regulatory action effectively and significantly attenuates cellular uptake of extracellular cystine, culminating in a consequential reduction of intracellular glutathione (GSH) biosynthesis and, accordingly, orchestrating the meticulous induction and marked potentiation of ferroptosis.

Based upon molecular mechanisms, a diverse array of pharmacologically anti-tumor drugs has been proactively developed and extensively employed to instigate ferroptosis in tumor cells. Such as Erastin, glutamic acid, sorafenib, and sulfasalazine, among others, achieve ferroptosis induction through the specific inhibition of System Xc- transporter functionality. This targeted inhibition effectively impedes the cellular uptake and influx of extracellular cystine, thereby indirectly suppressing GPX4 enzymatic activity and ultimately leading to the highly efficient induction of ferroptosis in tumor cells. In parallel, a distinct class of compounds, encompassing RSL3, ML162, FIN56, and FINO2, adopts an alternative yet convergent strategy by directly targeting the GPX4 protein molecule, exerting direct enzymatic inhibitory effects (26). These dual approaches, albeit mechanistically divergent, ultimately converge on the common outcome of efficiently and specifically inducing ferroptosis.

In summation, the precise modulation of the GPX4-centered signaling pathway represents a core intervention point and a pivotal therapeutic target for the accurate manipulation and regulation of ferroptosis initiation and progression. Future endeavors focused on the in-depth dissection of the molecular intricacies governing GPX4 and its upstream signaling regulatory pathways will undoubtedly lay a robust theoretical groundwork for the innovative development of oncotherapeutic strategies and furnish highly promising pharmacological targets.

### 2.2 The FSP1-CoQ10 pathway

FSP1, operating within the p53-mediated apoptosis pathway, is also recognized as p53-responsive gene (PRG) (27). Within the context of ferroptosis, FSP1 functions as an oxidoreductase of coenzyme Q10 (CoQ10) (28) (**Figure 1**). The N-terminal myristoylation of FSP1, a lipid acylation modification, serves to target FSP1 to the plasma membrane, thereby mediating NADH-dependent CoQ10 reduction, subsequently suppressing lipid peroxidation, and ultimately exerting an inhibitory effect on ferroptosis (29, 30). Of note, the mevalonate (MVA) pathway can also modulate ferroptosis progression through endogenous CoQ10 biosynthesis (31). In 2021, research conducted by Mao et al. revealed that dihydroorotate dehydrogenase (DHODH) can reduce CoQ10 within the inner mitochondrial membrane, thereby mitigating the occurrence of ferroptosis (32).

## 3 Metabolism in ferroptosis

### 3.1 The iron metabolism

Iron, as a fundamental trace element in the human body, participates in a variety of normal physiological and biochemical reactions, with dietary intake being its primary source (33). A fraction of iron in the body is stored in red blood cells as a reserve, while in peripheral tissues, iron is primarily stored in the form of ferritin and iron-sulfur clusters. Furthermore, a portion of iron within cells exists in the form of labile iron pools (LIP). Intracellular iron levels are precisely regulated and mainly depend on the absorption, storage, and discharge of iron.

Iron regulatory protein 1 (IRP-1) and iron regulatory protein 2 (IRP-2) are both key transcription factors involved in the regulation of iron metabolism; they can regulate the expression of transferrin (TF) and transferrin receptor 1 (TFR1), thereby influencing TF and TFR1-mediated iron transport from the extracellular to the intracellular space (34). Therefore, TF and TFR1 are also considered potential regulatory targets of IRP-1 and IRP-2. Ferroportin (FPN) is primarily responsible for regulating the efflux of excess intracellular iron (**Figure 1**), and FPN is mainly distributed on the surface of macrophages, hepatocytes, and absorptive enterocytes (35). Within this pathophysiological context, studies have found that liraglutide can reduce intracellular iron deposition by downregulating transferrin receptor 1 (TFR1) expression and upregulating Ferroportin-1 (FPN1) expression, thereby exerting an inhibitory effect on ferroptosis. Research by Wen et al. has also confirmed that autophagy can promote the occurrence of ferroptosis by degrading ferritin in fibroblasts and tumor cells. Overexpression of nuclear receptor coactivator 4 (NCOA4) can enhance autophagy-mediated ferritin degradation, thereby increasing the level of labile iron pools (LIP) within cells (36). It is noteworthy that excessive iron ions in the Fenton reaction can react with hydrogen peroxide to generate hydroxyl radicals with stronger oxidizing capacity, which in turn increases intracellular ROS levels, promotes lipid peroxidation, and ultimately triggers ferroptosis (37). Cancer cells, compared to normal cells, exhibit a higher dependency on iron and are more sensitive to the effects caused by iron metabolism disorders (38). In conclusion, targeting ferroptosis is considered a highly promising strategic direction in the field of cancer treatment.

### 3.2 The lipid metabolism

In homeostatic cellular physiology, the redox balance of lipids is meticulously maintained in a tightly controlled dynamic equilibrium. Lipid peroxidation is established as a hallmark biochemical event in ferroptosis (39). Accumulated ROS within cells can directly react with polyunsaturated fatty acids (PUFAs) in phospholipid bilayers, consequently and significantly altering membrane permeability, culminating in cellular demise (40). Acyl-CoA synthetase long-chain family member 4 (ACSL4) catalyzes the acylation of PUFAs to generate PUFA-CoA (polyunsaturated fatty acid acyl-CoA), which is subsequently re-esterified by lysophosphatidylcholine acyltransferase 3 (LPCAT3) to ultimately yield lipid peroxides (41, 42). Thus, modulation of ACSL4 and LPCAT3 expression or activity can demonstrably influence cellular susceptibility to ferroptosis (**Figure 1**) (43). Research by Yuan et al. has substantiated that genetic knockout of ACSL4 markedly inhibits RSL3-induced ferroptosis, with the inhibitory effect of GPX4-ACSL4 double knockout being even more pronounced (44).

Lipoxygenase (LOX) is capable of oxidizing PUFAs on cell membranes, thereby participating in the regulation of ferroptosis. Studies conducted by Ron et al. have revealed that LOX overexpression renders cells more susceptible to ferroptosis. However, LOX inhibitors have been validated as effective antioxidants capable of achieving the opposing outcome through the suppression of lipid peroxidation (45). In conclusion, lipid metabolism pathways play a central and pivotal role in ferroptosis; moreover, therapeutics designed to modulate lipid metabolism exhibit tremendous therapeutic potential in oncotherapeutic strategy.

## 4 Ferroptosis and immune cells

With the development of ferroptosis research, the iron dependency of immune cells has garnered increasing attention (46, 47). Factors that induce ferroptosis not only potentially trigger lipid peroxidation in immune cells but may also further compromise their functionality and viability (48). Conversely, immune cells, by altering their polarization states, can actively modulate iron metabolism within the tumor microenvironment, thereby inducing ferroptosis in tumor cells (49, 50).

### 4.1 T cell

GPX4 plays a crucial role in T cell function. *In vitro* experiments have demonstrated that GPX4-deficient T cells rapidly accumulate membrane lipid peroxides, thereby initiating ferroptosis and culminating in cell death (51). Conversely, the selenium-GPX4 axis effectively safeguards follicular helper T cells from ferroptosis damage, suggesting that targeted activation of this axis holds promise for enhancing T cell immune responses during infection and post-vaccination (52). Research has revealed that CD36-mediated fatty acid uptake by tumor-infiltrating CD8+ T cells is capable of inducing lipid peroxidation and ferroptosis (53), subsequently leading to diminished cytotoxic production and ultimately attenuating their anti-tumor efficacy (20). T cells also play a pivotal role in inducing ferroptosis in tumor cells during immunotherapy. A study elucidated that interferon-gamma (IFN-γ) released from CD8+ T cells can downregulate the expression levels of SLC3A2 and SLC7A11 on the tumor cell surface, thereby suppressing cystine uptake by tumor cells and ultimately promoting tumor cell lipid peroxidation and ferroptosis (54). Furthermore, T cell-derived IFN-γ can further stimulate ACSL4 activity and alter the tumor cell lipid profile, consequently increasing the incorporation of arachidonic acid (AA) into phospholipids enriched with C16 and C18 acyl chains, ultimately inducing immunogenic tumor ferroptosis (55).

Regulatory T cells (Treg cells) represent a critically important subset of cells in orchestrating anti-tumor immune responses (56). GPX4 functions to prevent Treg cells from undergoing lipid peroxidation and ferroptosis, thereby exerting a protective role in maintaining immune homeostasis and potentiating anti-tumor immunity. Mechanistic studies reveal that T cell receptor (TCR)/CD28 co-stimulation in GPX4-deficient Treg cells leads to aberrant accumulation of lipid peroxides and heightened iron toxicity; conversely, neutralization of lipid peroxides or blockade of iron bioavailability can effectively reverse the ferroptosis of GPX4-deficient Treg cells (57).

### 4.2 Macrophages

Macrophages represent a crucial subset of cells within the host immune system, typically differentiating into two functionally distinct phenotypes: classically activated (M1-type) macrophages and alternatively activated (M2-type) macrophages (58). Macrophages play a pivotal role in the regulation of iron homeostasis, achieving iron recycling through the phagocytosis of senescent or damaged erythrocytes (59). Indeed, the majority of iron elements required for physiological metabolism are derived from macrophage-mediated recycling following erythrophagocytosis (60). Studies have demonstrated that iron overload can upregulate the expression levels of M1-type macrophage marker proteins, such as IL-6, IL-1β, and CD40, while concurrently reducing the gene expression of the M2-type marker protein TGM2, thereby driving macrophage polarization towards the M1 phenotype (61). Mechanistic investigations have elucidated that the mechanism underlying iron overload-induced M1 polarization is intricately linked to elevated reactive oxygen species (ROS) production and upregulated p53 protein acetylation levels; conversely, reduction in ROS levels can significantly inhibit M1 polarization and p53 acetylation (62).

In cancer immunotherapy, the elimination of M2-type macrophages, or the reprogramming of M2-type macrophages towards an anti-tumorigenic M1 phenotype, represents a highly focused area of current research (63). Research reports have indicated that engineered exosome-like nanovesicles (eNVs-FAP) can effectively decrease the proportion of immunosuppressive cells – encompassing M2-like tumor-associated macrophages (M2-TAMs), myeloid-derived suppressor cells (MDSCs), and Tregs – within the tumor microenvironment (TME), thereby promoting tumor ferroptosis (64). Furthermore, studies have discovered that RRM2 inhibition can markedly promote M1-type macrophage polarization *in vitro* and *in vivo*, and suppress M2-type macrophage polarization progression. Further investigation has substantiated that the ferroptosis inhibitor ferrostatin-1 can effectively reverse the macrophage polarization effect mediated by RRM2 inhibition (65).

### 4.3 Natural killer cells

Natural killer cells (NK cells), as a crucial component of the innate immune system, exhibit diverse biological functions, encompassing anti-tumor immunity and anti-viral infection. Given the pivotal role of NK cells in immune regulation, numerous ongoing clinical trials are actively exploring NK cell-based immunotherapies, or evaluating the therapeutic value of monoclonal antibodies targeting NK cell immune checkpoints in cancer treatment (66).

The interplay between multiple components of ferroptosis and NK cells is increasingly gaining research attention. A recent study elucidated that the functional impairment of NK cells within the tumor microenvironment is closely associated with elevated levels of cell membrane lipid peroxidation. Notably, activation of the NRF2 antioxidant signaling pathway can effectively reverse the metabolic disorders and functional deficits of NK cells, ultimately demonstrating significantly enhanced anti-tumor activity in *in vivo* experimental models (67).

*In vitro* studies have indicated that the ferroptosis inducer Erastin is capable of inducing lipid peroxidation in the cell membrane, thereby promoting the proliferation and differentiation of human peripheral blood mononuclear cells (PBMCs), eventually directing differentiation towards B cells and NK cells (68). Furthermore, research has revealed that interferon-γ (IFN-γ) secreted by NK cells can significantly downregulate the mRNA and protein expression levels of SLC3A2 and SLC7A11, thus inducing ferroptosis in tumor cells (69). Of particular interest, research on the interplay between other types of tumor-infiltrating immune cells and ferroptosis remains relatively scarce, suggesting that this field may represent a highly promising avenue for future investigation in immuno-oncology.

## 5 Ferroptosis and tumor immunotherapy

Drug resistance and immune escape have become major bottlenecks that urgently need to be overcome in the field of cancer immunotherapy (70). The pivotal role of ferroptosis in cancer therapy is prompting researchers to re-examine and deeply analyze comprehensive cancer treatment strategies integrating radiotherapy, chemotherapy, and the immune system (71, 72). Conclusive evidence indicates that targeted therapeutic drugs, chemotherapeutic modalities, and even radiotherapy possess the potential to induce ferroptosis, thereby effectively enhancing the immunogenicity of tumor cells and promoting the infiltration of immune-active cells into the tumor microenvironment (73). The combined application of immunotherapy and ferroptosis-inducing strategies may contribute to a significant improvement in the clinical therapeutic benefits of immune checkpoint inhibitor (ICI) immunotherapy.

Inhibition of TYRO3 can promote tumor cell ferroptosis, enhance the sensitivity of resistant tumors to anti-PD-1 therapy, and drive the development of a pro-tumor microenvironment by reducing the M1/M2 macrophage ratio (73). Interferon-gamma (IFN-γ) regulates the expression of SLC3A2, SLC7A11, and ACSL4, subsequently inducing immunogenic ferroptosis in tumor cells. Furthermore, IFN-γ plays a critical role in the synergistic effects of immunotherapy and radiotherapy. IFN-γ derived from immunotherapy-activated CD8+ T cells and radiotherapy-activated ATM independently inhibits SLC7A11, ultimately leading to reduced cystine uptake, elevated tumor lipid peroxidation levels, and the occurrence of ferroptosis (18). Targeting ferroptosis and immunotherapy therapy can enhance the anti-tumor therapeutic efficacy.

Certain drugs that induce ferroptosis also exhibit modulatory effects on the tumor immune system. Examples include Erastin, Lenvatinib, Cisplatin, and other agents within this category. A study revealed that the ferroptosis inducer Erastin did not elicit cell death in human PBMCs. Conversely, Erastin-induced lipid peroxidation, counterintuitively, promoted the proliferation and differentiation of human PBMCs into B lymphocytes and natural killer cells by suppressing the expression of the bone morphogenetic protein (BMP) family. Consequently, in-depth investigation into the combined application of such ferroptosis-inducing drugs with immunotherapeutic agents holds significant potential value for enhancing cancer treatment outcomes. A list of ferroptosis drugs with immunomodulatory effects is detailed in **Table 1**.

**Table 1:** Drugs and molecules induce ferroptosis in cancers and effects on immunity.

Drug name	Target	Tumor	Effect on immunity	References
Erastin	System Xc-	non-small cell lung cancer	promotes human PBMC proliferation and differentiation into B cells and natural killer cells	(68, 74)
Lenvatinib	NRF2	hepatoma carcinoma cell	decreased the proportion of monocytes and macrophages population and increased that of CD8 T cell populations	(75, 76)
Artesunate	ROS	hepatoma carcinoma cell	enhances T-cell-mediated antitumor activity	(77)
Cisplatin	GSH	non-small cell lung cancer	increases PD-L1 expression	(78, 79)
Metformin	miR-324-3p/GPX4	breast cancer	increases CTL activity by reducing the stability and membrane localization of programmed death ligand-1 (PD-L1)	(80, 81)

Several novel nanomedicines are used for the treatment of tumors by inducing tumor ferroptosis while modulating immunity. Emerging novel nanomedicines are under investigation for tumor therapy, leveraging the induction of tumor ferroptosis in conjunction with immunomodulation. The ultrasmall single-crystal Fe nanoparticles (bcc-USINPs) could efficiently induce tumor cell ferroptosis and promote the maturation of dendritic cells, and trigger the adaptive T cell response (82). In combination with programmed death-ligand 1 (PD-L1) immune checkpoint blockade immunotherapy, bcc-USINP-mediated ferroptosis therapy significantly potentiated the immune response and fostered robust immunological memory. Fe3O4 -SAS@PLT are built from sulfasalazine (SAS)-loaded mesoporous magnetic nanoparticles (Fe3O4) and platelet (PLT) membrane camouflage which triggered a ferroptosis cell death through inhibiting the glutamate-cystine antiporter system Xc- pathway and it also efficiently repolarize macrophages from immunosuppressive M2 phenotype to antitumor M1 phenotype (83). New nanodrugs offer a novel cancer treatment strategies by ferroptosis-based immunotherapy. These innovative nanodrugs present a straightforward, safe, and efficacious novel strategy for ferroptosis-based immunotherapy.

## 6 Conclusion

Malignancies exhibit a high incidence and mortality, with certain tumor types characterized by a notably unfavorable prognosis. Immunotherapy represents an efficacious modality for tumor treatment by modulating the host immune response; however, its effectiveness is frequently attenuated by tumor immune escape mechanisms. Consequently, the exploration of novel therapeutic avenues is imperative. The induction of ferroptosis in neoplastic cells presents a promising strategy for tumor eradication. Numerous clinically approved drugs, including sorafenib, lorazepam, artemisinin, cisplatin, haloperidol, and gemcitabine, have demonstrated the capacity to induce tumor ferroptosis, either as monotherapy or in combination regimens. Furthermore, iron metabolism and ferroptosis are recognized to play a crucial role in the functionality of tumor-infiltrating lymphocytes. It is also established that specific targeted therapies, chemotherapy, and radiotherapy can induce ferroptosis, augment the immunogenicity of tumor cells, and promote immune cell infiltration into the tumor microenvironment. Moreover, certain innovative nanomedicines are capable of inducing tumor ferroptosis while concurrently modulating the host immune response. Nevertheless, considering the nascent stage of related clinical investigations, definitive conclusions remain constrained. It is anticipated that with larger cohort studies and advancements in research methodologies, novel pharmaceuticals and therapeutic strategies will emerge imminently, predicated upon a comprehensive elucidation of the interplay between ferroptosis and immunotherapy and a meticulous investigation and refinement of the underlying mechanisms of ferroptosis.
